# Supplementary figures and images for: Comparison of two one-piece acrylic foldable intraocular lenses: Short-term change in axial movement after cataract surgery and its effect on refraction
Source: PLoS One. 2022 Aug 30;17(8):e0273431. doi: 10.1371/journal.pone.0273431 (PMC9426912; doi:10.1371/journal.pone.0273431)

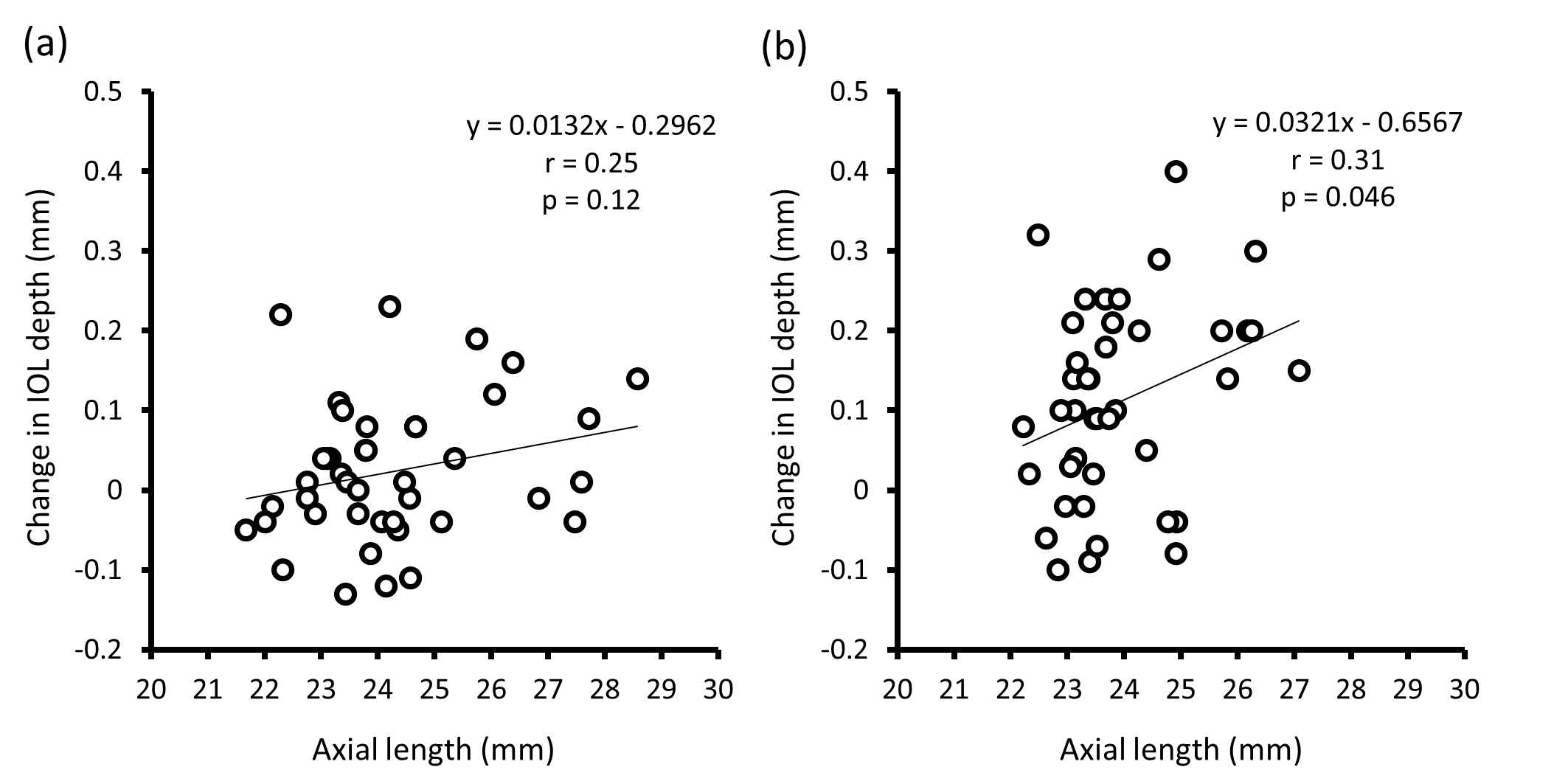

Supplement: S1 Fig — (TIF) [file pone.0273431.s001.tif]
