# Supplementary material for: Comparison of two one-piece acrylic foldable intraocular lenses: Short-term change in axial movement after cataract surgery and its effect on refraction
Source: PLoS One. 2022 Aug 30;17(8):e0273431. doi: 10.1371/journal.pone.0273431 (PMC9426912; doi:10.1371/journal.pone.0273431)
Supplement: S1 File — (DOCX) [file pone.0273431.s002.docx]

**Supporting Information**

**Method:**

Zemax OpticStudio (ZOS) was used to evaluate the refractive errors from the change in postoperative lens position (the difference between 4 days postop and 1 month postop).

All ocular biometric parameters were obtained from preoperative measurement data and assumed to be constant in order to not interfere with the lens position variability.

The procedures were as follows:

1. Cornea anterior radius curvature was calculated by 337.5/PreOp(AveK).
2. Cornea posterior radius curvature was assumed to be constant for all subjects, and defined as 6.5 mm.
3. Cornea center thickness was assumed to be constant for all subjects, and defined as 550 mm.
4. Cornea and aqueous refractive indices were defined as 1.376 and 1.336, respectively.
5. IOL design and refractive indices were given by the manufacturers.
6. Axial power was obtained by PreOp(AL).
7. Lens positions were determined using the measurement results at 4 days and 1 month.
8. Back focal length (the distance from the posterior IOL to the retina) was then calculated using the aforementioned distances.

All the information was inputted into the ZOS Lens Data Editor, and ZOS calculated the Effective Focal Length (EFL) and axial Longitudinal Spherical Aberration (LSA0) (Supplemental figure).

The refractive error was calculated using the following formula

SE = -(1000/EFL – 1000/(EFL-LSA0))


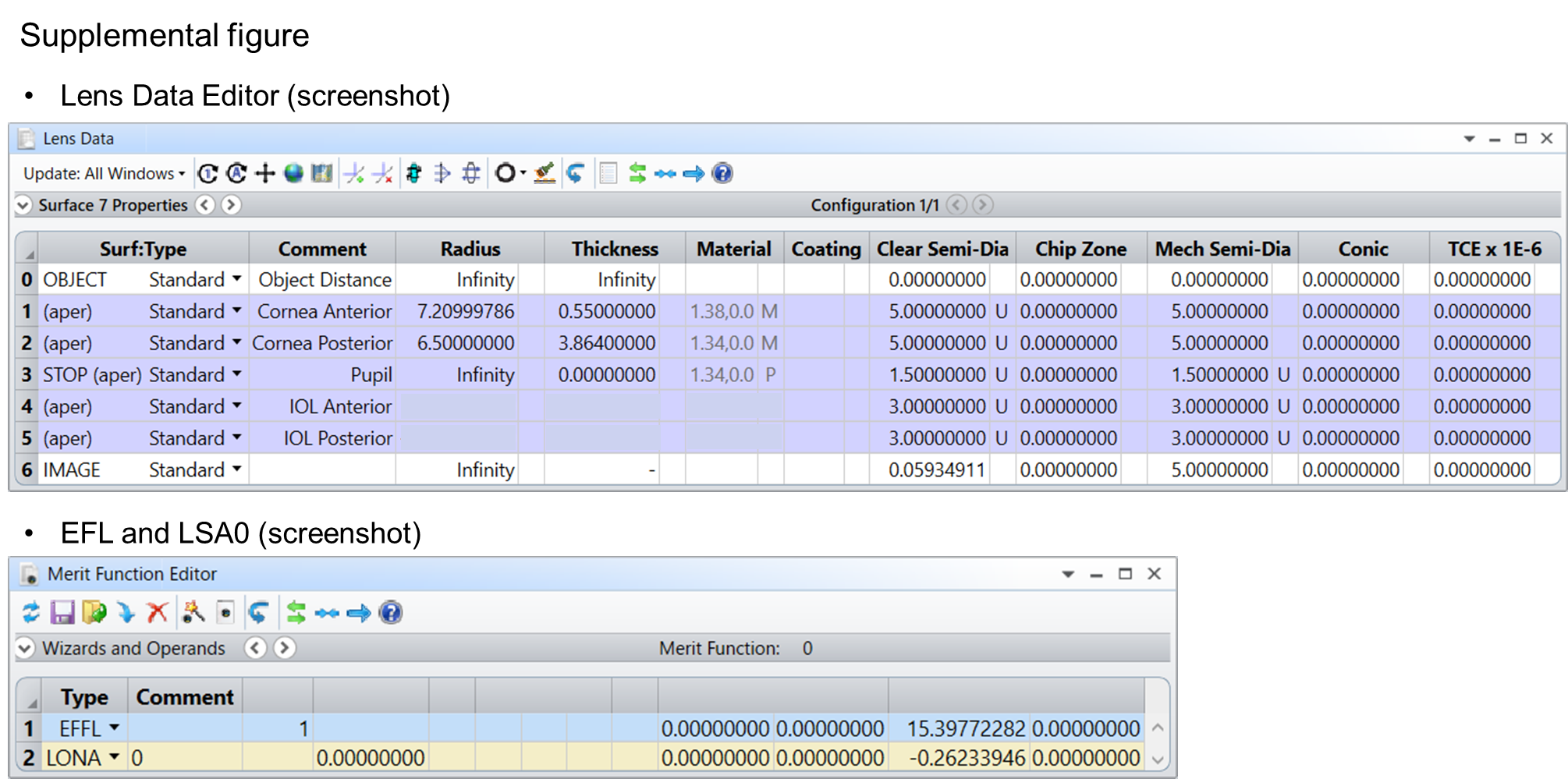


**Raw data of ZOSModel.zmx:**

MODE SEQ

NAME

PFIL 0 0 0

LANG 0

UNIT MM X W X CM MR CPMM

FLOA

ENVD 35 1 1

GFAC 0 0

GCAT SCHOTT

RAIM 0 2 1 1 0 0 0 0 0 1

PUSH -2.7131892210931558e-05 0 0 0 0 0

SDMA 0 1 0

OMMA 1 1

FTYP 0 0 1 1 0 0 0

ROPD 2

HYPR 0

PICB 1

XFLN 0 0 0 0 0 0 0 0 0 0 0 0

YFLN 0 0 0 0 0 0 0 0 0 0 0 0

FWGN 1 1 1 1 1 1 1 1 1 1 1 1

VDXN 0 0 0 0 0 0 0 0 0 0 0 0

VDYN 0 0 0 0 0 0 0 0 0 0 0 0

VCXN 0 0 0 0 0 0 0 0 0 0 0 0

VCYN 0 0 0 0 0 0 0 0 0 0 0 0

VANN 0 0 0 0 0 0 0 0 0 0 0 0

WAVM 1 0.54607399999999995 1

WAVM 2 0.55000000000000004 1

WAVM 3 0.55000000000000004 1

WAVM 4 0.55000000000000004 1

WAVM 5 0.55000000000000004 1

WAVM 6 0.55000000000000004 1

WAVM 7 0.55000000000000004 1

WAVM 8 0.55000000000000004 1

WAVM 9 0.55000000000000004 1

WAVM 10 0.55000000000000004 1

WAVM 11 0.55000000000000004 1

WAVM 12 0.55000000000000004 1

WAVM 13 0.55000000000000004 1

WAVM 14 0.55000000000000004 1

WAVM 15 0.55000000000000004 1

WAVM 16 0.55000000000000004 1

WAVM 17 0.55000000000000004 1

WAVM 18 0.55000000000000004 1

WAVM 19 0.55000000000000004 1

WAVM 20 0.55000000000000004 1

WAVM 21 0.55000000000000004 1

WAVM 22 0.55000000000000004 1

WAVM 23 0.55000000000000004 1

WAVM 24 0.55000000000000004 1

PWAV 1

POLS 1 0 1 0 0 1 0

GLRS 3 0

GSTD 0 100.000 100.000 100.000 100.000 100.000 100.000 0 1 1 0 0 1 1 1 1 1 1

NSCD 100 500 0 0.001 5 9.9999999999999995e-07 0 0 0 0 0 0 1000000 0 2

COFN QF "COATING.DAT" "SCATTER_PROFILE.DAT" "ABG_DATA.DAT" "PROFILE.GRD"

COFN COATING.DAT SCATTER_PROFILE.DAT ABG_DATA.DAT PROFILE.GRD

SURF 0

COMM Object Distance

TYPE STANDARD

FIMP

CURV 0.0 0 0 0 0 ""

HIDE 0 0 0 0 0 0 0 0 0 0

MIRR 2 0

SLAB 9

DISZ INFINITY

DIAM 0 0 0 0 1 ""

MEMA 0 0 0 0 1 ""

POPS 0 0 0 0 0 0 0 0 1 1 1 1 0 0 0 0

SURF 1

COMM Cornea Anterior

TYPE STANDARD

FIMP

CURV 1.386962962962962342E-01 0 0 0 0 ""

HIDE 0 0 0 0 0 0 0 0 0 0

MIRR 2 0

SLAB 1

DISZ 0.55000000000000004

GLAS ___BLANK 1 0 1.3759999999999999 0 0 0 0 0 0 0

DIAM 5 1 0 0 1 ""

MEMA 5 0 0 0 1 ""

POPS 0 0 0 0 0 0 0 0 1 1 1 1 0 0 0 0

FLAP 0 5 0

SURF 2

COMM Cornea Posterior

TYPE STANDARD

FIMP

CURV 1.538461538461538547E-01 0 0 0 0 ""

HIDE 0 1 0 0 0 0 0 0 0 0

MIRR 2 0

SLAB 2

DISZ 3.8639999999999999

GLAS ___BLANK 1 0 1.3360000000000001 0 0 0 0 0 0 0

DIAM 5 1 0 0 1 ""

MEMA 5 0 0 0 1 ""

POPS 0 0 0 0 0 0 0 0 1 1 1 1 0 0 0 0

FLAP 0 5 0

SURF 3

COMM Pupil

STOP

TYPE STANDARD

FIMP

CURV 0.0 0 0 0 0 ""

HIDE 0 1 0 0 0 0 0 0 0 0

MIRR 2 0

SLAB 3

DISZ 0

GLAS ___BLANK 2 2 1.3360000000000001 0 0 0 0 0 0 0

DIAM 1.5 1 0 0 1 ""

MEMA 1.5 1 0 0 1 ""

POPS 0 0 0 0 0 0 0 0 1 1 1 1 0 0 0 0

FLAP 0 1.5 0

SURF 4

COMM IOL Anterior

TYPE STANDARD

FIMP

CURV 5.263157894736841813E-02 0 0 0 0 ""

HIDE 0 0 0 0 0 0 0 0 0 0

MIRR 2 0

SLAB 4

DISZ 0.69999999999999996

GLAS ___BLANK 1 0 1.5 0 0 0 0 0 0 0

DIAM 3 1 0 0 1 ""

MEMA 3 1 0 0 1 ""

POPS 0 0 0 0 0 0 0 0 1 1 1 1 0 0 0 0

FLAP 0 3 0

SURF 5

COMM IOL Posterior

TYPE STANDARD

FIMP

CURV -5.263157894736841813E-02 0 0 0 0 ""

HIDE 0 1 0 0 0 0 0 0 0 0

MIRR 2 0

SLAB 5

DISZ 17.274000000000001

GLAS ___BLANK 2 2 1.3360000000000001 0 0 0 0 0 0 0

DIAM 3 1 0 0 1 ""

MEMA 3 1 0 0 1 ""

POPS 0 0 0 0 0 0 0 0 1 1 1 1 0 0 0 0

FLAP 0 3 0

SURF 6

TYPE STANDARD

FIMP

CURV 0.0 0 0 0 0 ""

HIDE 0 0 0 0 0 0 0 0 0 0

MIRR 2 0

SLAB 8

DISZ 0

DIAM 0.040796963281374721 0 0 0 1 ""

MEMA 5 0 0 0 1 ""

POPS 0 0 0 0 0 0 0 0 1 1 1 1 0 0 0 0

EFFL 0 1 0 0 0 0 0 0 0 0

LONA 0 0 0 0 0 0 0 0 0 0

TOL TOFF 0 0 0 0 0 0 0 0

MNUM 1 1

MOFF 0 1 "" 0 0 0 1 1 0 0.0 "" 0
