# Supplementary material for: Comparison of two one-piece acrylic foldable intraocular lenses: Short-term change in axial movement after cataract surgery and its effect on refraction
Source: PLoS One. 2022 Aug 30;17(8):e0273431. doi: 10.1371/journal.pone.0273431 (PMC9426912; doi:10.1371/journal.pone.0273431)
Supplement: Attachment — Submitted filename: PONE-D-22-01418 Response to Reviewers.docx [file pone.0273431.s003.docx]

**Manuscript ID:** PONE-D-22-01418

**Title:** Comparison of two one-piece acrylic foldable intraocular lenses: Short-term change in axial movement after cataract surgery and its effect on refraction

**Point-by-Point Responses**

**All reviewers:**

Previously each IOL evaluated in this study was described by the product name. Based on the reviewers’ point, we are now referencing the products with descriptors “with hinges or without hinges” in lieu of product names to better clarify the argument of this manuscript.

**Reviewer #1**:

1. How did you assess you case number?

**Response:** We performed the sample size power calculation. At the end of the method section, we mentioned as follows; The sample size was calculated to detect a difference in error of 0.10 mm between 2 groups; with a signiﬁcance level of 5%, a statistical power of 80%, and assuming standard deviation (SD) to 0.1 mm, 34 eyes were required. Page 7, line 132.

2. Was the CASIA measurement performed only once in each case?

**Response:** Thank you for this comment. The CASIA was performed three times in each visit and data is exported as the mean value of three measurements. We have added the explanation in the Methods section. Page6, line 111.

3. Was it performed by one examiner? Or more?

**Response:** Thank you for this comment. Yes, the CASIA was performed by one experienced ophthalmic technician. We have added clarification to the Methods section. Page 6, line 111.

4. Could you please provide ZEMAX simulation results or table as supplementary material?

**Response:** Thank you for this comment. In Table 2, we have already described the simulation results of refraction change associated with change in IOL position, those were estimated using OpticStudio 16.5 Sp5 (Zemax, LLC.), the industry standard optical system design software; however, the representation of the parameter was not clear. Therefore, we have changed “Estimated refractive error associated with change in IOL depth” to “Refractive change associated with change in IOL depth” and have added the phrase of “^C^ calculated by the OpticStudio software” in the footnote of Table 2.

**Reviewer #2:**

Methods: When the study was retrospectively conducted, it is unlikely that it took 15 months. Rather patients which had surgery in this timespan were included.

**Response:** Thank you for this comment. As the reviewer pointed out, this was a retrospective, observational, comparative, single-center study of all patients who had undergone uncomplicated cataract surgery at the National Hospital Organization, Tokyo Medical Center, Tokyo, Japan, between December 2015 and February 2017. We corrected the sentence at the beginning of the Methods section. Page 5, line 74.

Can you explain in short how the ZEMAX software works?

**Response:** The Zemax software, the official name is "OpticStudio" [Zemax, LLC.], is the premier optical design software used by engineers around the globe for 30 years. We have corrected the name of the software in the manuscript. We can estimate the theoretical postoperative refractive change that is caused by postoperative axial movement of IOL by using the OpticStudio software (see the attached screenshot of OpticStudio software). For example, if one eye showed 0.2 mm forward shift of IOL between postoperative 4 days and 1 month, the refractive changes caused by the change in IOL position are affected by the IOL power, corneal power, and axial length. Eventually, we can calculate the estimated refractive change associated with change in IOL depth. The results of these simulations using the OpticStudio was shown in Table 2 as “Refractive change associated with change in IOL depth (D)^c^.” “C” is explained in the footnote of Table 2.

Did you use the post-OP Cataract module of the Casia 2 software?

**Response:** Yes, we used the post-op cataract module of the Casia2, which gave us the postoperative aqueous depth, corneal thickness, and anterior chamber depth as well.

Why did the authors include 42 eyes when only 34 were required according to their sample size calculation? Due to the retrospective nature of the study, no drop out correction is necessary.

**Response:** Thank you for this comment. Before the current study started, we had a data set of 42 eyes for each group. When we proposed this study, we performed a sample size calculation to confirm whether the subject number was enough for the investigation of the topic.

In this revision, we have randomly selected 34 eyes from 42 eyes, and analyzed the data. We have validated this analysis three times. As the result, every data set demonstrated significant difference shown in the table below.

| Comparison of IOL depth between IQ Toric and XY-1 | | | |
| --- | --- | --- | --- |
| n = 34 | IQ Toric | XY-1 | P value |
| 1^st^ | 0.014 ± 0.076 | 0.098 ± 0.127 | 0.0015 |
| 2^nd^ | 0.023 ± 0.090 | 0.096 ± 0.126 | 0.0077 |
| 3^rd^ | 0.029 ± 0.018 | 0.129 ± 0.018 | 0.0002 |
| 34 eyes were randomly selected from 42 eyes | | | |

In addition, as we mentioned the detail in the next question below, once we use the SD of 0.11, the power calculation tells us that 41 subjects are needed. Based on these calculation result, the data set of 42 subjects seems to be appropriate for this investigation.

Why did the authors assume 0.1mm as a SD? There are a lot of studies available with SD`s for axial movement of IOLs.

**Response:** Thank you for this constructive comment. To determine the SD of the change in IOL position to perform the power calculation, we referred to the article published by Hayashi K, et al. (doi:10.1016/j.ajo.2020.05.031.). Given that the SD of the change in IOL position in the paper was 0.11mm, we should have use 0.11 mm for SD. If we performed sample size power analysis to detect a difference in error of 0.1 mm between two groups; with a signiﬁcance level of 5%, a statistical power of 80%, and assuming standard deviation (SD) to 0.11 mm, 41 eyes were required. Based on these data, we concluded that 42 subjects are appropriate to investigate the postoperative change in IOL depth. We have edited the sentence related to power sample analysis at the end of the Methods section. Page 6, line 132.

Since you study axial movement of the eye, you should clearly state which viscoelastics were used during the conduct of the study. Did the surgeon also remove the OVD from behind the lens? I ask this because one of the IOLs was a toric IOL, usually a surgeon removes the OVD behind the lens very thoroughly to avoid postoperative IOL rotation due to remaining viscoelastic.
Did the surgeon remove viscoelastics for both groups under the same manner? Or were the same viscoealstics used? This could affect postoperative axial stabilization within the first weeks.

**Response:** Thank you for this insightful comment. I have added the information of OVD. Additionally, the surgeon removed the same OVD with the same manner for both groups. Briefly, the I/A tip is inserted behind the IOL optic and the posterior chamber is directly irrigated and cleaned. I have added the explanations of these procedures in the Methods section. Page 5, line 94.

How was the postoperative refraction (MRSE) assessed at 4 days and 1 month? Objective or subjective refraction?

**Response:** Thank you for this comment. Subjective MRSE was assessed at 4 days and 1 month.

I miss a detailed description of both IOLs in the methods section (overall diameter, optic diameter, capsular bag angle, haptic resilience, and other characteristics)

**Response:** Thank you for this comment. We have added the information of both IOLs in the Methods section of the manuscript with the photograph of both IOLs. Both of the IOLs made of acrylic material have 13.0 mm overall diameter, 6.0 mm optic diameter, and planar-haptic design, whereas IQ Toric IOL has a specific flexible hinge design. We have added the information of each IOL in the Methods section (page 5, line 92). There is no available information of haptic resilience.

Results:
It’s a little bit confusing that you present change in IOL depth and actual axial movement. What`s the difference between these two variables? You should describe this in detail in the methods. Are the results absolute values? Since the IOL can move forward and backwards? This is a very essential point.

**Response:** Thank you for this constructive comment. Whereas the change in IOL depth is the numerical value, the actual axial movement is the absolute value. As the reviewer pointed out the definitions of these parameters are a little confusing, we have replaced “actual axial movement” with “absolute axial movement” in Table 2.

I would also report the range for the IOL depths and/or IOL axial movements [min;max] or seeing Boxplots of the results to get a better feeling were all the results are located.

**Response:** Thank you for this constructive comment. I have added the data of [min:max] in Table 2 and have made the graph including all dots as Figure 3 (see below).

**Fig 3**. **Fig 3. Mean (± standard deviation) change in IOL depth between 4 days and 1 month after cataract surgery.** IOL, intraocular lens; W/O, without; Student *t*-test, ****p* < 0.001

Discussion:
What do the authors mean by “constricted” and “straight” haptics, what exactly is the difference between the haptics of these two IOLs? A figure would be good for better understandability.

**Response:** We appreciate this valuable comment. We have added the photographs of IOLs as Fig 1 that emphasizes the difference of the haptics structure.

**Fig 1. Optic and haptic configurations of (a) IOL with hinges and (b) IOL without hinges**. While both of IOLs have planar haptics designs with a 0-degree angle, the haptics of the IOL with hinges (c) are constricted (i.e. flexible hinge design, black arrows) and the haptics of the IOL without hinges (d) are straight.

The overall discussion is well written.
I miss a single point: Since both of the IOLs show pretty much the same design, what may be the main reason or multiple reasons for this difference between the IOLs in axial movement?

**Response:** Thank you for this constructive comment. Both of IOLs have planar-haptic design, whereas IQ Toric IOL has a specific flexible hinge design (Fig 1. C, black arrows). Based on an in vitro experiment (IOL compression test), the planar haptics with a flexible hinge design could minimize axial forces and allow the IOLs to remain planar when compressed. ^13^ Although the axial stability of IOLs has been proved in vitro, there was less clinical data. We believe that the difference in haptic configurations would be the main factor that provides the postoperative stability.

13. Lane S, Collins S, Das KK, et al. Evaluation of intraocular lens mechanical stability. J Cataract Refract Surg 2019;45:501–506.

Another important point: was IOL movement correlated with axial length? It has been shown in earlier studies, that shorter eyes (or shorter preoperative ACDs) are more prone to axial IOL movement postoperatively. (Schartmüller et al, JRS 2021) (Schartmüller et al ESCRS 2019 Paris)

**Response:** Thank you for this constructive comment. We have confirmed the relationship between axial length and axial IOL movement, indicating that longer eyes are more prone to axial IOL movement postoperatively in only XY-1 (IOL without hinges) group, which is the opposite result of Schartmüller et al, JRS 2021. There are several differences between our study and Schartmüller et al, such as the observation period and the type of IOL. We assumed that longer eye tends to have larger bag, and as such IOLs can more easily to move in the bag postoperatively. At least, the result shown as S1 Fig might also indicate that IQ Toric IOLs (IOL with hinges) are more stable. We have added this result to the Discussion section and added supplemental figure 1. Page12, line 194.

**S1 Fig.** Correlations between axial length and the changes in IOL depth between 4 days and 1 month after cataract surgery: (a) IOL with hinges and (b) IOL without hinges.

**Reviewer #3:**

The authors present an interesting manuscript about two different IOL (haptic) designs and their axial stability in the postoperative course. Results are set in context with refractive changes. Interestingly, the authors chose a toric lens and a nontoric lens, which leads to differences in the centration of the haptics, still I like the study design.
While I think that overall, the manuscript is very well composed, I do see a few open questions: With Aqueous depth as main criterion, the authors reported significant changes in posterior radial curvature but failed to report changes in CCT and in ACD. Changes in posterior corneal curvature might be a sign of swelling of the corneal endothelium, certain levels of Descemet striae and swelling are not seldom after cataract surgery. AQD should not be described without ACD and CCT.

**Response:** Thank you for providing this insightful comment. As the reviewer recommended, we have analyzed the changes in CCT and ACD. Whereas there was no significant difference in change in CCT between 2 groups (p = 0.31), the change in ACD was significantly smaller in IQ Toric (IOL with hinges) group than XY-1 (IOL without hinges) group, which is the same as the result of IOL depth (i.e. postoperative AQD). We have added these results in Table 2. Subsequently, the change in ACD was significantly correlated with the refractive changes in XY-1 group (r = 0.63, *p* < 0.0001), but there was no significant difference in IQ Toric group (r = 0.21, *p* = 0.20); this result is also the same as the change in AQD.

What is lacking is a discussion about the ideal timeframe for lens constant optimization, as a shift of the IOL and refraction obviously influence constant optimization if refractive results for certain types of IOLs are analyzed too early.

**Response:** Thank you for this valuable comment. We agree with the reviewer’s comment. Therefore, we have added the following discussion in the Discussion section: The postoperative stability of IOL position is one important factor for maintaining refractive stability. Page 13, line 230.

The purpose of the current study is to investigate the postoperative IOL axial movement, not improve the prediction error of the IOL power calculation formulas. Even though there are a lot of IOL formulas, we can say that at least 1 month or more after surgery is required to evaluate the postoperative refraction as many studies reported.

I would think that an autorefractometer is influenced by the Abbe number of the IOL and a diffractive form. We see systematically differing results from subjective refraction that differ for various IOLs. I don’t necessarily advise to change to ARF. Furthermore, repeatability has proven better for us for subjective refraction than for pseudophakic objective refraction. It is of note that we actually have 0.125 Dpt steps for subjective refraction (although not for a phoropter) that can be used if 0.25 Dpt seems to be too imprecise.

**Response:** We truly appreciate your comments. As you recommended, we would like to have 0.125 D steps for subjective refraction, which would give us more promising results in this field. I have added this recommendation in the Discussion section. Page14, line 241.
