# Supplementary material for: Comparison of two one-piece acrylic foldable intraocular lenses: Short-term change in axial movement after cataract surgery and its effect on refraction
Source: PLoS One. 2022 Aug 30;17(8):e0273431. doi: 10.1371/journal.pone.0273431 (PMC9426912; doi:10.1371/journal.pone.0273431)
Supplement: Attachment — Submitted filename: @PONE-D-22-01418 Response to Reviewers2.docx [file pone.0273431.s004.docx]

**Manuscript ID:** PONE-D-22-01418

**Title:** Comparison of two one-piece acrylic foldable intraocular lenses: Short-term change in axial movement after cataract surgery and its effect on refraction

**Point-by-Point Response 2**

**Reviewer #1**:

The authors have properly addressed all comments. The manuscript is now improved in all its part. I suggest to send the exact ZEMAX table as Supplementary materials.

**Response:** Thank you for this constructive comment. At the reviewer’s suggestion, we have added supporting information, which includes an exact ZEMAX table based on the “zmx” file and screenshots of the Lens Data Editor and Effective Focal Length (EFL) and axial Longitudinal Spherical Aberration (LSA0). Page 6, line 121.

**Supporting Information**

**Method:**

Zemax OpticStudio (ZOS) was used to evaluate the refractive errors from the change in postoperative lens position (the difference between 4 days postop and 1 month postop).

All ocular biometric parameters were obtained from preoperative measurement data and assumed to be constant in order to not interfere with the lens position variability.

The procedures were as follows:

1. Cornea anterior radius curvature was calculated by 337.5/PreOp(AveK).
2. Cornea posterior radius curvature was assumed to be constant for all subjects, and defined as 6.5 mm.
3. Cornea center thickness was assumed to be constant for all subjects, and defined as 550 mm.
4. Cornea and aqueous refractive indices were defined as 1.376 and 1.336, respectively.
5. IOL design and refractive indices were given by the manufacturers.
6. Axial power was obtained by PreOp(AL).
7. Lens positions were determined using the measurement results at 4 days and 1 month.
8. Back focal length (the distance from the posterior IOL to the retina) was then calculated using the aforementioned distances.

All the information was inputted into the ZOS Lens Data Editor, and ZOS calculated the Effective Focal Length (EFL) and axial Longitudinal Spherical Aberration (LSA0) (Supplemental figure).

The refractive error was calculated using the following formula

SE = -(1000/EFL – 1000/(EFL-LSA0))

**Raw data of ZOSModel.zmx:**

MODE SEQ

NAME

PFIL 0 0 0

LANG 0

UNIT MM X W X CM MR CPMM

FLOA

ENVD 35 1 1

GFAC 0 0

GCAT SCHOTT

RAIM 0 2 1 1 0 0 0 0 0 1

PUSH -2.7131892210931558e-05 0 0 0 0 0

SDMA 0 1 0

OMMA 1 1

FTYP 0 0 1 1 0 0 0

ROPD 2

HYPR 0

PICB 1

XFLN 0 0 0 0 0 0 0 0 0 0 0 0

YFLN 0 0 0 0 0 0 0 0 0 0 0 0

FWGN 1 1 1 1 1 1 1 1 1 1 1 1

VDXN 0 0 0 0 0 0 0 0 0 0 0 0

VDYN 0 0 0 0 0 0 0 0 0 0 0 0

VCXN 0 0 0 0 0 0 0 0 0 0 0 0

VCYN 0 0 0 0 0 0 0 0 0 0 0 0

VANN 0 0 0 0 0 0 0 0 0 0 0 0

WAVM 1 0.54607399999999995 1

WAVM 2 0.55000000000000004 1

WAVM 3 0.55000000000000004 1

WAVM 4 0.55000000000000004 1

WAVM 5 0.55000000000000004 1

WAVM 6 0.55000000000000004 1

WAVM 7 0.55000000000000004 1

WAVM 8 0.55000000000000004 1

WAVM 9 0.55000000000000004 1

WAVM 10 0.55000000000000004 1

WAVM 11 0.55000000000000004 1

WAVM 12 0.55000000000000004 1

WAVM 13 0.55000000000000004 1

WAVM 14 0.55000000000000004 1

WAVM 15 0.55000000000000004 1

WAVM 16 0.55000000000000004 1

WAVM 17 0.55000000000000004 1

WAVM 18 0.55000000000000004 1

WAVM 19 0.55000000000000004 1

WAVM 20 0.55000000000000004 1

WAVM 21 0.55000000000000004 1

WAVM 22 0.55000000000000004 1

WAVM 23 0.55000000000000004 1

WAVM 24 0.55000000000000004 1

PWAV 1

POLS 1 0 1 0 0 1 0

GLRS 3 0

GSTD 0 100.000 100.000 100.000 100.000 100.000 100.000 0 1 1 0 0 1 1 1 1 1 1

NSCD 100 500 0 0.001 5 9.9999999999999995e-07 0 0 0 0 0 0 1000000 0 2

COFN QF "COATING.DAT" "SCATTER_PROFILE.DAT" "ABG_DATA.DAT" "PROFILE.GRD"

COFN COATING.DAT SCATTER_PROFILE.DAT ABG_DATA.DAT PROFILE.GRD

SURF 0

COMM Object Distance

TYPE STANDARD

FIMP

CURV 0.0 0 0 0 0 ""

HIDE 0 0 0 0 0 0 0 0 0 0

MIRR 2 0

SLAB 9

DISZ INFINITY

DIAM 0 0 0 0 1 ""

MEMA 0 0 0 0 1 ""

POPS 0 0 0 0 0 0 0 0 1 1 1 1 0 0 0 0

SURF 1

COMM Cornea Anterior

TYPE STANDARD

FIMP

CURV 1.386962962962962342E-01 0 0 0 0 ""

HIDE 0 0 0 0 0 0 0 0 0 0

MIRR 2 0

SLAB 1

DISZ 0.55000000000000004

GLAS ___BLANK 1 0 1.3759999999999999 0 0 0 0 0 0 0

DIAM 5 1 0 0 1 ""

MEMA 5 0 0 0 1 ""

POPS 0 0 0 0 0 0 0 0 1 1 1 1 0 0 0 0

FLAP 0 5 0

SURF 2

COMM Cornea Posterior

TYPE STANDARD

FIMP

CURV 1.538461538461538547E-01 0 0 0 0 ""

HIDE 0 1 0 0 0 0 0 0 0 0

MIRR 2 0

SLAB 2

DISZ 3.8639999999999999

GLAS ___BLANK 1 0 1.3360000000000001 0 0 0 0 0 0 0

DIAM 5 1 0 0 1 ""

MEMA 5 0 0 0 1 ""

POPS 0 0 0 0 0 0 0 0 1 1 1 1 0 0 0 0

FLAP 0 5 0

SURF 3

COMM Pupil

STOP

TYPE STANDARD

FIMP

CURV 0.0 0 0 0 0 ""

HIDE 0 1 0 0 0 0 0 0 0 0

MIRR 2 0

SLAB 3

DISZ 0

GLAS ___BLANK 2 2 1.3360000000000001 0 0 0 0 0 0 0

DIAM 1.5 1 0 0 1 ""

MEMA 1.5 1 0 0 1 ""

POPS 0 0 0 0 0 0 0 0 1 1 1 1 0 0 0 0

FLAP 0 1.5 0

SURF 4

COMM IOL Anterior

TYPE STANDARD

FIMP

CURV 5.263157894736841813E-02 0 0 0 0 ""

HIDE 0 0 0 0 0 0 0 0 0 0

MIRR 2 0

SLAB 4

DISZ 0.69999999999999996

GLAS ___BLANK 1 0 1.5 0 0 0 0 0 0 0

DIAM 3 1 0 0 1 ""

MEMA 3 1 0 0 1 ""

POPS 0 0 0 0 0 0 0 0 1 1 1 1 0 0 0 0

FLAP 0 3 0

SURF 5

COMM IOL Posterior

TYPE STANDARD

FIMP

CURV -5.263157894736841813E-02 0 0 0 0 ""

HIDE 0 1 0 0 0 0 0 0 0 0

MIRR 2 0

SLAB 5

DISZ 17.274000000000001

GLAS ___BLANK 2 2 1.3360000000000001 0 0 0 0 0 0 0

DIAM 3 1 0 0 1 ""

MEMA 3 1 0 0 1 ""

POPS 0 0 0 0 0 0 0 0 1 1 1 1 0 0 0 0

FLAP 0 3 0

SURF 6

TYPE STANDARD

FIMP

CURV 0.0 0 0 0 0 ""

HIDE 0 0 0 0 0 0 0 0 0 0

MIRR 2 0

SLAB 8

DISZ 0

DIAM 0.040796963281374721 0 0 0 1 ""

MEMA 5 0 0 0 1 ""

POPS 0 0 0 0 0 0 0 0 1 1 1 1 0 0 0 0

EFFL 0 1 0 0 0 0 0 0 0 0

LONA 0 0 0 0 0 0 0 0 0 0

TOL TOFF	0	0	0	0	0 0 0 0

MNUM 1 1

MOFF	0	1 "" 0 0 0 1 1 0 0.0 "" 0

**Reviewer #3:**

I didn’t view this as critical in my first review, but as both other reviewers noticed this point I have to agree that the description of how OpticStudio was used is kind of lacking in this study. Apparently the authors used a simulation model with 7 rays? Which model eye was chosen? Did the authors use full aperture raytracing or the paraxial simplification form? Was the individual pupil size and asphericity considered? Considering 2 different IOL designs were used was IOL geometry data known? At least all the actual simulation details used for this analysis should be provided in the materials section.

**Response:** Thank you for this constructive comment. As the reviewer pointed out, we have added the details related to how to analyze the data using ZEMAX software in the Method section as follows (page 6, line 115):

The calculations with OpticStudio were performed in the paraxial form. Therefore, the number of rays and aperture size did not contribute to the calculated results. Although asphericity affects the spherical results when comparing 4 days and 1 month spherical differences, to simplify the analysis, asphericity can be removed from the calculation of spherical differences. The eye model was built from the patients' biometry data including the cornea power, aqueous depth, and AL (refer to the Supporting Information).

Additionally, we have added the following “Supporting Information” file to describe the details of how to analyze the data with the OpticStudio.

**Supporting Information**

**Method:**

Zemax OpticStudio (ZOS) was used to evaluate the refractive errors from the change in postoperative lens position (the difference between 4 days postop and 1 month postop).

All ocular biometric parameters were obtained from preoperative measurement data and assumed to be constant in order to not interfere with the lens position variability.

The procedures were as follows:

9. Cornea anterior radius curvature was calculated by 337.5/PreOp(AveK).
10. Cornea posterior radius curvature was assumed to be constant for all subjects, and defined as 6.5 mm.
11. Cornea center thickness was assumed to be constant for all subjects, and defined as 550 mm.
12. Cornea and aqueous refractive indices were defined as 1.376 and 1.336, respectively.
13. IOL design and refractive indices were given by the manufacturers.
14. Axial power was obtained by PreOp(AL).
15. Lens positions were determined using the measurement results at 4 days and 1 month.
16. Back focal length (the distance from the posterior IOL to the retina) was then calculated using the aforementioned distances.

All the information was inputted into the ZOS Lens Data Editor, and ZOS calculated the Effective Focal Length (EFL) and axial Longitudinal Spherical Aberration (LSA0) (Supplemental figure).

The refractive error was calculated using the following formula

SE = -(1000/EFL – 1000/(EFL-LSA0))

**Raw data of ZOSModel.zmx:**

MODE SEQ

NAME

PFIL 0 0 0

LANG 0

UNIT MM X W X CM MR CPMM

FLOA

ENVD 35 1 1

GFAC 0 0

GCAT SCHOTT

RAIM 0 2 1 1 0 0 0 0 0 1

PUSH -2.7131892210931558e-05 0 0 0 0 0

SDMA 0 1 0

OMMA 1 1

FTYP 0 0 1 1 0 0 0

ROPD 2

HYPR 0

PICB 1

XFLN 0 0 0 0 0 0 0 0 0 0 0 0

YFLN 0 0 0 0 0 0 0 0 0 0 0 0

FWGN 1 1 1 1 1 1 1 1 1 1 1 1

VDXN 0 0 0 0 0 0 0 0 0 0 0 0

VDYN 0 0 0 0 0 0 0 0 0 0 0 0

VCXN 0 0 0 0 0 0 0 0 0 0 0 0

VCYN 0 0 0 0 0 0 0 0 0 0 0 0

VANN 0 0 0 0 0 0 0 0 0 0 0 0

WAVM 1 0.54607399999999995 1

WAVM 2 0.55000000000000004 1

WAVM 3 0.55000000000000004 1

WAVM 4 0.55000000000000004 1

WAVM 5 0.55000000000000004 1

WAVM 6 0.55000000000000004 1

WAVM 7 0.55000000000000004 1

WAVM 8 0.55000000000000004 1

WAVM 9 0.55000000000000004 1

WAVM 10 0.55000000000000004 1

WAVM 11 0.55000000000000004 1

WAVM 12 0.55000000000000004 1

WAVM 13 0.55000000000000004 1

WAVM 14 0.55000000000000004 1

WAVM 15 0.55000000000000004 1

WAVM 16 0.55000000000000004 1

WAVM 17 0.55000000000000004 1

WAVM 18 0.55000000000000004 1

WAVM 19 0.55000000000000004 1

WAVM 20 0.55000000000000004 1

WAVM 21 0.55000000000000004 1

WAVM 22 0.55000000000000004 1

WAVM 23 0.55000000000000004 1

WAVM 24 0.55000000000000004 1

PWAV 1

POLS 1 0 1 0 0 1 0

GLRS 3 0

GSTD 0 100.000 100.000 100.000 100.000 100.000 100.000 0 1 1 0 0 1 1 1 1 1 1

NSCD 100 500 0 0.001 5 9.9999999999999995e-07 0 0 0 0 0 0 1000000 0 2

COFN QF "COATING.DAT" "SCATTER_PROFILE.DAT" "ABG_DATA.DAT" "PROFILE.GRD"

COFN COATING.DAT SCATTER_PROFILE.DAT ABG_DATA.DAT PROFILE.GRD

SURF 0

COMM Object Distance

TYPE STANDARD

FIMP

CURV 0.0 0 0 0 0 ""

HIDE 0 0 0 0 0 0 0 0 0 0

MIRR 2 0

SLAB 9

DISZ INFINITY

DIAM 0 0 0 0 1 ""

MEMA 0 0 0 0 1 ""

POPS 0 0 0 0 0 0 0 0 1 1 1 1 0 0 0 0

SURF 1

COMM Cornea Anterior

TYPE STANDARD

FIMP

CURV 1.386962962962962342E-01 0 0 0 0 ""

HIDE 0 0 0 0 0 0 0 0 0 0

MIRR 2 0

SLAB 1

DISZ 0.55000000000000004

GLAS ___BLANK 1 0 1.3759999999999999 0 0 0 0 0 0 0

DIAM 5 1 0 0 1 ""

MEMA 5 0 0 0 1 ""

POPS 0 0 0 0 0 0 0 0 1 1 1 1 0 0 0 0

FLAP 0 5 0

SURF 2

COMM Cornea Posterior

TYPE STANDARD

FIMP

CURV 1.538461538461538547E-01 0 0 0 0 ""

HIDE 0 1 0 0 0 0 0 0 0 0

MIRR 2 0

SLAB 2

DISZ 3.8639999999999999

GLAS ___BLANK 1 0 1.3360000000000001 0 0 0 0 0 0 0

DIAM 5 1 0 0 1 ""

MEMA 5 0 0 0 1 ""

POPS 0 0 0 0 0 0 0 0 1 1 1 1 0 0 0 0

FLAP 0 5 0

SURF 3

COMM Pupil

STOP

TYPE STANDARD

FIMP

CURV 0.0 0 0 0 0 ""

HIDE 0 1 0 0 0 0 0 0 0 0

MIRR 2 0

SLAB 3

DISZ 0

GLAS ___BLANK 2 2 1.3360000000000001 0 0 0 0 0 0 0

DIAM 1.5 1 0 0 1 ""

MEMA 1.5 1 0 0 1 ""

POPS 0 0 0 0 0 0 0 0 1 1 1 1 0 0 0 0

FLAP 0 1.5 0

SURF 4

COMM IOL Anterior

TYPE STANDARD

FIMP

CURV 5.263157894736841813E-02 0 0 0 0 ""

HIDE 0 0 0 0 0 0 0 0 0 0

MIRR 2 0

SLAB 4

DISZ 0.69999999999999996

GLAS ___BLANK 1 0 1.5 0 0 0 0 0 0 0

DIAM 3 1 0 0 1 ""

MEMA 3 1 0 0 1 ""

POPS 0 0 0 0 0 0 0 0 1 1 1 1 0 0 0 0

FLAP 0 3 0

SURF 5

COMM IOL Posterior

TYPE STANDARD

FIMP

CURV -5.263157894736841813E-02 0 0 0 0 ""

HIDE 0 1 0 0 0 0 0 0 0 0

MIRR 2 0

SLAB 5

DISZ 17.274000000000001

GLAS ___BLANK 2 2 1.3360000000000001 0 0 0 0 0 0 0

DIAM 3 1 0 0 1 ""

MEMA 3 1 0 0 1 ""

POPS 0 0 0 0 0 0 0 0 1 1 1 1 0 0 0 0

FLAP 0 3 0

SURF 6

TYPE STANDARD

FIMP

CURV 0.0 0 0 0 0 ""

HIDE 0 0 0 0 0 0 0 0 0 0

MIRR 2 0

SLAB 8

DISZ 0

DIAM 0.040796963281374721 0 0 0 1 ""

MEMA 5 0 0 0 1 ""

POPS 0 0 0 0 0 0 0 0 1 1 1 1 0 0 0 0

EFFL 0 1 0 0 0 0 0 0 0 0

LONA 0 0 0 0 0 0 0 0 0 0

TOL TOFF	0	0	0	0	0 0 0 0

MNUM 1 1

MOFF	0	1 "" 0 0 0 1 1 0 0.0 "" 0

Regarding the answer to my own comments on IOL constants optimization:
“The purpose of the current study is to investigate the postoperative IOL axial movement, not improve the prediction error of the IOL power calculation formulas.”
Well, why else are we interested in axial IOL movement, if not for right (right lens constant) and stable (good IOL design) refractive results?
Anyways, “we can say that at least 1 month or more after surgery is required to evaluate the postoperative refraction as many studies reported” is a sufficient statement, this doesn’t have to go into an in-depth analysis of every IOL calculation formula.

**Response:** We appreciate this valuable comment, and totally agree with your comment.

Methods:
I think the statement “the industry standard optical system design” sounds a bit like advertisement, can this be omitted?

**Response:** Thank you for this comment. We have deleted the part in the manuscript as recommended.

I figure the refraction lane length was standardized? Which lane length was used?

Response: Thank you for this comment. CDVA was measured at 5 meters. Therefore, we have added this information in the Method section. Page 7, line 124.
